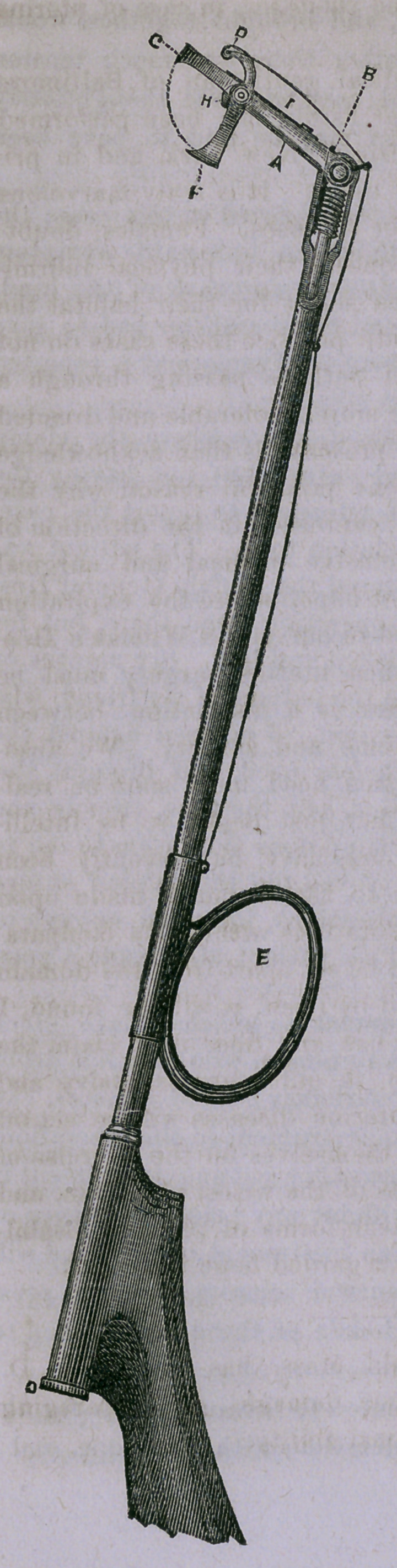# Vesico-Vaginal Fistula

**Published:** 1867-12

**Authors:** C. C. F. Gay

**Affiliations:** One of the Surgeons of the Buffalo General Hospital


ART. II.—*Vesico-Vaginal Fistula.* BY C. C. F. GAY, M. D., *one of the Surgeons of the Buffalo General Hospital.*

After having read over what has been written by us in a former paper upon the use of the anæsthetic in surgical operations, involving the female genital organs, we have feared that the false impression may have been conveyed of the indispensibility of the use of chloroform. We hasten therefore to correct such impression.

It is a remarkable fact that during abnormal condition of the vagina and uterus, there is but little sensibility, especially in the mucus coverings of these organs; hence chloroform is never absolutely necessary in operations involving them. Chloroform being used chiefly and generally to relieve pain, and exceptionally as a pacificator to the patient, and the operations being for the most part painless operations, chloroform is of course of but little use otherwise than as a placebo.

The absence of sensibility of the genital organs when in their abnormal state is most apparent in procidentia uteri. The condition which this turn is designed to represent, is that condition which exists when the os protrudes beyond and outside the vulva from half an inch to four inches, more or less. The constant attrition, friction or rubbing together of the uterine mucous membrane with the vaginal mucous membrane; or to be more definite in form of expression I should say that the constant attrition of the mucous membrane lining the vagina, with that portion of mucous membrane reflected upon the neck of the uterus, producing an inverted vagina, thickens and roughens the membrane to that extent that the process of denudation with the scissors is scarcely felt by the patient until the final cut is made near the neck of the bladder; therefore I repeat, that chloroform is by no means essential in what is usually denominated uterine surgery, and may in the majority of cases be entirely dispensed with.

The only value which I am able to see, that attaches to the report of individual cases, consists in illustration of some difficult point in the case. In the operation for fistula the mode of proceeding in any two cases is not precisely the same. No two cases present the same points of difficulty. As in human faces, no two that might be selected are alike in contour. So likewise in vesico-vaginal fistula, no two cases which might be selected will present the same points of resemblance; hence in operating in a hundred cases the surgeon must be prepared to vary, although but slightly perhaps, his mode of operation in every succeeding case. It is because of this invariability of the lesion and non-uniform method of procedure, that any considerable value attaches to report of different cases of a malady essentially alike in all its general features.

Mrs. M. presented herself for examination shortly after confinement, complaining that she could not retain her urine. Up to this time she was totally ignorant of the nature of her malady. On inquiring into the history of her case we learned that at her secand confinement the head of the child become impacted, and so remained for two or three hours; pains had nearly ceased, and delivery was completed by the use of forceps. Soon thereafter she felt her water dribbling away, and over which she had no control. More than a year passed before this patient came under our observation again. She had suffered so much with this constant annoyance and with the consequent abrasion and inflammation of the parts with which the flow of urine had come in contact, that she readily assented to an operation, leaving for her home with the promise that she would put herself immediately under the necessary preliminary treatment for operation. At the expiration of three weeks the operation was made. She was placed in the semi-prone position of Sims, and chloroform administered. The index finger of left hand was introduced within the fistula, on which was raised the upper border of the fistula; the mucous membrane denuded with the curved scissors with the greatest possible ease. More trouble was experienced in denuding the lower border, which was seized with the tenaculum and denuded with scissors, consuming much more time than was necessary in preparing the opposite border. This done, it was found that the two opposing surfaces could not be brought well together unless so much tension was used that sutures would be liable to tear out, and for the moment it seemed that the operation might prove a failure, but at this juncture discovering several small fibrous bands which appeared to exert an influence in keeping the scarified surfaces from apposition, these bands were snipped, and a slight incision made upon one side of the fistula at a distance of about three-quarters of an inch, when I was happy to find that the tension was entirely removed. But this process of snipping and making but slight incision caused a hemorrhage which was extremely troublesome. Repeated applications of sol. ferri persulphates, diluted with four parts of water, failing to arrest the hemorrhage, common vinegar was used with success, which almost induces me to recommend this agent as a hæmostatic superior to any other for use in uterine surgery. The parts were now brought together by five interrupted silver sutures. The needle used was three-quarters of an inch in length, armed with a long silk loop. After the silk loop had been passed the silver wire was attached to the loop and drawn through just far enough for the short end of the wire to be bent around the long extremity of the wire, and the long extremity was then given into the hands of an assistant, and this process was continued until the remaining four wires in like manner passed through the two borders of the fistula. The needles were held and passed by the needle forceps. Then beginning with the first suture introduced the wire was seized by the long forceps, and the long extremity of the wire cut to equal length with the shorter extremity, and the wire twister bent to a convenient angle, applied over both strands, and sufficient tension made to unite the denuded surfaces, the wire was twisted down upon the holder when the instrument was slipped from the suture, and the wire cut off, leaving it about one inch in length. Proceeding in like manner with the remaining sutures it was found that the sides of the fistula were nicely approximated.

The operation lasted about two hours, but need not have lasted over an hour and a half had no hemorrhage occurred. This hemorrhage was in consequence of an error of mine, and might, I think, have been provided against before the final operation. It were just as easy to have learned before the operation that small bands held the parts from coaptation, as to have learned this fact during the operation, and should, I think, have been divided when the patient was in process of preliminary preparation for the operation, then hemorrhage would not have come in as a perturbing element to delay the progress of the operation. Tenacula hooked into either side the fistula border, and brought together, would enable one to ascertain the fact whether there was much tension or none whatever. This simple and easy trial was thoughtlessly omitted, and therefore the operation may be said to have been commenced prematurely.

There is nothing further of note to be reported in this case; the patient made a good recovery without an untoward symptom beyond what may usually be anticipated in any case of the kind. The urethra became very irritable, and Sims' catheter had to give place to the ordinary gum-elastic catheter. The patient is reported to me now as entirely well.

It was my purpose to report, in brief, a case where the clamp was used instead of the interrupted suture, but not having the unqualified authority of the medical attendant to report the case, will make only a remark or two in relation to it. The patient was a German, 28 years of age. She entered the Buffalo General Hospital for the purpose of having the operation done there, but for some reason or other became dissatisfied and left. Six months or a year afterwards the woman became the patient of my friend, who invited me to assist him in the operation. I advised against the use of the clamp, but it was nevertheless used, and brought the parts denuded beautifully in coaptation, but the time, trouble and patience required in adjusting the clamp does not seem to me to justify its use, when experience has proved the interrupted suture adequate to fill all the required indications, provided enough of them are used in any given case. This patient also made a good recovery.

I have recently devised an instrument to which I have given the name Intra Uterine Scissors. Any person who may not deem himself an adept in the use of the ordinary curved scissors, can be greatly aided with the use of this instrument in this operation. It will be found on experience that no other cutting instrument is required, and any one can use it without any prior experience or practice. I hardly think, however, an instrument of this kind will come into general use, because the curved scissors appear at present to answer every purpose in the hands of those accustomed to their use.

This instrument is herewith presented to the profession for the first time for their impartial consideration, with the hope, and I

may say, expectation of having contributed in our humble way, something useful to the armamentaria of our art. Four to six pair of scissors, differing in curve, are usually required by the surgeon who attempts this operation which I have but cursorily discussed, while this single instrument alone is sufficient to accomplish the entire operation of cutting. The accompanying diagram will give the reader an intelligent idea of the mechanism of the instrument. But a section of the handle is here represented on account of the space which is wanting to show it. The cutting edge of the instrument is about three-eights of an inch in length.

The lever "D" is worked by means of a fine wire passing through guides to the slide "E," which receives the index finger, while the milled head of the inside rod "C" is turned by the thumb; thus by means of the combination of screw, slide and ratchet at "B," setting the arm "A" at any required angle from the shaft. The jaws, "F" and "G," are relatively like those of an ordinary steel-trap, except that the sharp edge of "F" shuts *over* that of "G," so as to bite cleanly and entirely out whatever they may be required to remove. These jaws keep apart when the wire is loose, by means of a spring "I" acting upon an excentric at "H."

This instrument may be found possibly useful for dividing the pedicle of a polypus, or for denuding the mucous membrane well up in the *cul-de-sac*, in case of uterine displacement, etc.

I am credibly informed by a medical gentleman of Baltimore that this operation for vesico-vaginal fistula has been performed by Dr. Emmet of the Woman's Hospital, New York, and in private practice, two hundred and fifty times. It is truly marvelous from whence come so large a number of cases. Females, doubtless, to a great extent, religiously conceal their physical infirmities, especially when those infirmities select for their habitat the genital organs, consequently in private practice these cases do not come to light, the victims of them perhaps passing through a world of trouble and physical torture more intolerable and dreaded than death itself, preferring to suffer present ills than acknowledge their disability. This is at least one powerful reason why the charities of communities should be exercised in the direction of establishing institutions for the exclusive medical and surgical treatment of the female. May we not hope, before the expiration of another decade, to see establi shed in our city a Woman's Hospital? The time has now come when uterine surgery must be separated from general surgery; there is a distinction between them as broad as that between medicine and surgery. We absolutely need a division of labor, and this need must soon be realized. The field of uterine surgery has just begun to be intelligently cultivated; implements for use have but recently been placed in our hands at all adequate to the demands made upon them, and as we go on with our explorations within this comparatively new field of labor, which is to be set apart from the domain of operative surgery, to be cultivated by itself, it will be found, I trust, that the vast area, which ever has, and does now, claim the undivided energies of the profession, is quite too extensive and large, and that the department of uterine diseases will be set off by themselves as quite sufficient in themselves for the exercise of all the higher intellectual endowments of the wisest physician, and to cope successfully with these protean forms of physical disability, the studies of a life-time may be regarded none too much.

---

Dr. Anthony O'Reilly, of Springfield, Mass., has sued Dr. F. D. Mueller, of the same city, for $5,000 damages, for disparaging remarks touching O'Reilly's professional ability.

## Figures and Tables

**Figure f1:**